# Exploring the* Urtica dioica* Leaves Hemostatic and Wound-Healing Potential

**DOI:** 10.1155/2017/1047523

**Published:** 2017-10-17

**Authors:** Karama Zouari Bouassida, Sana Bardaa, Meriem Khimiri, Tarek Rebaii, Slim Tounsi, Lobna Jlaiel, Mohamed Trigui

**Affiliations:** ^1^Laboratory of Biopesticides, Center of Biotechnology of Sfax, University of Sfax, P.O. Box 1177, 3018 Sfax, Tunisia; ^2^Laboratory of Pharmacology, Faculty of Medicine of Sfax, Road Majida Boulila, 3028 Sfax, Tunisia; ^3^Laboratory of Histopathology, Faculty of Medicine of Sfax, Road Majida Boulila, 3028 Sfax, Tunisia

## Abstract

The present paper investigated the efficiency of* Urtica dioica (U. dioica)* on hemostatic and wound healing activities.* U. dioica* leaf extracts were evaluated for their antibacterial and antioxidant effects as well as their flavonoid and polyphenol content. The hydroethanolic extract (EtOH-H_2_OE), showing the most potent antibacterial and antioxidant activities* in vitro*, thanks to its flavonoid and polyphenol richness, was selected for hemostatic and wound healing evaluation. Twenty-four rats completing full-thickness wounds were split into four groups. The wounds were topically treated with saline solution, glycerol, “CICAFLORA,” and* U. dioica* EtOH-H_2_OE (50 *µ*L/mm^2^) until day 11. The wound healing effect was assessed by macroscopic, histological, and biochemical parameters. Rats treated with EtOH-H_2_OE showed fast wound closure (92.39%) compared to the control animals (60.91%) on the 11th day of wounding (*P* < 0.01). Histopathological and biochemical explorations showed full epidermal regeneration and an improvement of the hydroxyproline content in the* U. dioica* EtOH-H_2_OE treated rats. Analysis of fatty acids and sterols by GC-MS showed the presence of unsaturated fatty acids and a high concentration of lupeol known for their involvement in reepithelialization. These results prove the efficiency of* U. dioica* EtOH-H_2_OE in wound healing and supported its traditional use.

## 1. Introduction

The extraction and characterization of bioactive molecules from plants have opened new horizons to the development of pharmacological targets to reduce the marked adverse effects of chemical agents. Wound care medicinal plants have been in use since ancient civilizations. Plant extracts are well known in dermopharmacy for their abilities to promote wound healing via different molecular pathways such as the antioxidant and the antibacterial activities [1].

Wound healing is essential for the restoration of disturbed skin anatomical continuity. It involves different overlapping phases including inflammation, wound contraction, and reepithelialization with neovascularization [2]. Reactive oxygen species (ROS) play a major role in the management of the normal wound-healing process and harmfully affect cells and tissues [3]. Antioxidant products may lead to control wound oxidative stress and promote wound healing by shortening the healing time span [4].

Several attempts have been made to find drugs in traditional medicine to promote the healing of skin lesions.* U. dioica*, often called “nettle” or “stinging nettle,” is a herbaceous perennial flowering plant belonging to Urticaceae family and growing in the temperate zones of Asia, America, North Africa, and Europe [5]. Some scientists have studied the chemical composition of* U. dioica* and reported that its leaves contained a wide variety of chemical constituents such as minerals, vitamins, amino acids, flavonoids, sterols, phenolics, and fatty acids, which have beneficial effects on human health [6–9]. GC-MS analysis of* U. dioica *methanolic leaf extract showed the presence of cinnamic acid, coumarins, and homovanillic acid as phenolic compounds. The HPLC analysis of the extract also evidenced the presence of abundant *β*-carotenes and chlorophyll [10].

*U. dioica* was frequently used by humans for many medicinal purposes. In the home remedies, the nettle has been used as a source of medicinal preparations. Thanks to its antihemorrhagic effect, the powdered leaf extract was traditionally used to reduce nose bleeding and excessive menstrual flow [10]. This plant has been used for the treatment of rheumatism, arthritis, anemia, and prostate diseases in the folk medicine [11]. Several clinical and experimental studies investigated the* U. dioica* and suggested that nettle herb has some pharmacological properties such as anti-inflammatory [12], antibacterial, antioxidant, hypoglycemia [13], and antiviral activities [14].* U. dioica* has a long use record in the external treatment of skin problems. Nevertheless, no published work was undertaken justifying its wound-healing effect.

It was reported that the wound-healing efficiency requires the integration of many biological activities such as antioxidant and antibacterial properties of a plant extract [1]. This work therefore aimed to (i) explore the* in vivo* wound-healing activity of the Tunisian* U. dioica* with reference to its antioxidant and antimicrobial effects; (ii) characterize the phytochemical composition of the used extract by GC-MS; and (iii) analyze the relationship between its chemical composition and bioactivities.

## 2. Materials and Methods

### 2.1. Plant Drug Extraction

*U. dioica* fresh leaves were collected from Sfax (Tunisia, 35°14′58.36′′ N, +11°7′17.75′′ E) in February 2015 and the authentication was achieved agreeing to the flora of Tunisia. The plant was identified by the Pr. Mohamed Chaieb, botanist at the University of Science (Sfax, Tunisia) and a voucher specimen (LBPes UD 02.15) was deposited in the herbarium of the Laboratory of Biopesticides of the Centre of Biotechnology of Sfax.

The* U. dioica* dried leaves (100 g) were clipped into small parts with a blender and were macerated in ethanol-water (8 : 2, v/v) for 48 h to yield EtOH-H_2_OE. Then the extract was filtered using Whatman paper. The dried hydroethanolic crude extract (20 g) was sequentially partitioned into two solvents with increasing polarity:* n*-hexane and ethyl acetate. The organic solvents were evaporated at reduced pressure (Rotary Evaporator Buchi R-200, Switzerland) and the water fraction was lyophilized. Three fractions were thus obtained: hexanic fraction (*n*-Hex-F), ethyl acetate fraction (EtOAc-F), and water fraction (W-F) of* U. dioica*.

The stock solutions were kept at 4°C in the dark until a further analysis.

### 2.2. Gas Chromatography-Mass Spectrometry (GC-MS) Analysis of Phytosterols and Fatty Acids

#### 2.2.1. Saponification and Extraction

*U. dioica* EtOH-H_2_OE was saponified with 50 ml methanolic potassium hydroxide (2 N) for 1 h (under reflux) and then extracted three times with 100 ml hexane. The solvent was removed and concentrated in 1 ml* n*-hexane prior to the GC-MS analysis of terpenes and sterols. For the fatty acids analysis, using the* N*,O-bis(trimethylsilyl)trifluoroacetamide (BSTFA) a step of silylation was used prior to GC-MS analysis. BSTFA (200 *μ*l), 200 *μ*l extract of* U. dioica *EtOH-H_2_O in acetonitrile, and 50 *μ*l of pyridine were mixed and mechanically shaken for 2 min. The sample was placed in a water bath, at 80°C for 60 min, and then analyzed by GC-MS.

#### 2.2.2. GC-MS Identification

An Agilent 6890N Network GC system (Agilent Technologies) equipped with HP-5MS fused silica capillary column (30 m × 0.25 mm i.d. × 0.25 *μ*m film thickness) supplied by Agilent and coupled to a mass selective detector (MSD5973, ionization voltage 70 eV; all Agilent, Santa Clara, France) was used. Helium was used as a carrier gas at 1 ml/min flow rate. The GC oven temperature was held at 60°C for 2 min and then programmed to rise from 60 to 300°C at a rate of 5°C/min. The split/splitless injector (splitless mode) temperature was set to 280°C. The components were identified by careful examination of fragmentation patterns [15] and spectral data obtained from the Wiley and NIST libraries. Determination was carried out in duplicate.

### 2.3. Total Phenolic Content Determination

Total phenolic content was determined using the Folin-Ciocalteu method adapted to a microscale described by Waterman and Mole [16]. Gallic acid was used as a standard. The absorbance was measured at 760 nm and the results were expressed as mg of gallic acid equivalent per g of dry weight (mg GAE/g). Tests were performed in triplicate for each extract.

### 2.4. Determination of Total Flavonoids Content

The extracts total flavonoid content was assessed by the aluminum chloride spectrophotometric method [17]. The mixture absorbance was measured at 430 nm. The result of flavonoid content was expressed as mg of Quercetin equivalent per g dry weight (mg QE/g). Tests were performed in triplicate for each extract.

### 2.5. *In Vitro* Antioxidant Testing Assay

The free radical-scavenging activity and the capacity of the hydroalcoholic extract and derived fractions to ovoid *β*-carotene bleaching were measured, respectively, in triplicate using the methods described by Kirby and Schmidt [18] and Pratt [19]. The ascorbic acid and the Butylated hydroxyanisole (BHT) were taken as references.

### 2.6. Antibacterial Activity

The* U. dioica *organic extracts antibacterial effects were verified against two Gram-positive (*Staphylococcus aureus* ATCC 25923 and* Bacillus subtilis *ATCC 14579) and two Gram-negative (*Escherichia coli *ATCC 25922 and* Pseudomonas aeruginosa* ATCC 9027) bacteria. The bacterial strains were cultivated in Mueller-Hinton agar (MH) (Oxoid Ltd., UK) at the appropriated temperature for each strain for 24 hours. For the antibacterial activity, a freshly inoculum was prepared from isolated strains. Culture were maintained on Muller-Hinton agar slants at 4°C as stock cultures and subcultured once in 2 weeks.

The antimicrobial screening was performed using the agar well diffusion method according to Güven et al. [20] in triplicate. Minimum inhibitory concentrations (MIC) of* U. dioica* extracts were determined according to Eloff [21]. Microdilution test was performed in sterile 96-well microplates using 100 *μ*L/well. The final extracts concentrations in each well ranged from 0.156 to 10 mg/mL. The MIC was the lowest concentration of extracts at which the microorganism does not demonstrate visible growth after incubation. As an indicator of bacterial growth, 25 *μ*l of p-iodonitrotetrazolium chloride INT (0.5 mg/mL) dissolved in sterile water was added to the wells and incubated at 37°C for 30 min.

## 3. *In Vivo* Wound-Healing Activity Study

### 3.1. Preparation of* U. dioica* Extract

The EtOH-H_2_OE (yield = 20.6%) was dissolved at a concentration of 10% in sterile solution of glycerol and stirred until a homogeneous ointment was obtained. A dosage of 50 *μ*l/mm^2^ of the* U. dioica* extract from this ointment was topically administered on the wound.

### 3.2. Animals

Wistar rats (160–180 g) of either sex were obtained from Central Pharmacy of Tunisia (SIPHAT, Tunisia). The animals were housed under standard laboratory conditions at a temperature of 22 ± 2°C. They were kept in individual cages with food and water ad libitum. All experiments were conducted in accordance with the European Community guidelines (EEC directive of 1986; 86/609/EEC) for the care and use of laboratory animals and approved by the Committee of Animal Ethics (Directive 2001–2133) issued by the University of Sfax Tunisia.

### 3.3. Excision Wound Model

The backs of twenty-four rats were sterilized and shaved under anaesthesia (ketamine 100 mg/Kg body weight) before circular wound creation. Full thickness of predetermined dorsal region of rats was then carefully cut [22]. The period of epithelialization and wound contraction were noted. Wound contraction was measured as contraction rate each 2 days after wound induction. A specimen sample of tissue from the healed wounds was collected for histopathological examination.

### 3.4. Wound-Healing Activity

Animals with a mean weight of 160 g were split into four groups (*n* = 5): Group I: rats were just cleaned with saline solution (control group), Group II: rats were treated with glycerol (control group), Group III: rats were treated with a reference drug “CICAFLORA cream” (positive control), and Group IV: wounds were treated with 50 *μ*L/mm^2^ of EtOH-H_2_OE of* U. dioica*.

The treatments were topically applied and carried out each two days using sterilized compresses immediately after wound induction till the first group completely healed.

### 3.5. Bleeding Time in Rats

Adult Wistar rats of both sexes were divided into 3 groups (*n* = 6). The tip of the tail of each rat was cut with a scalpel blade to cause bleeding. As soon as the animal began to bleed a stopwatch was started simultaneously with the application of the test substance on the cut. The control group clipped tails were immersed in distilled water or normal saline solution. The tested group clipped tails were dipped in the hydroalcoholic extract of* U. dioica*. All the tails were vertically positioned on top of the blotting paper to wipe off blood. Stopwatch was stopped and bleeding time was recorded as soon as bleeding ended [23].

## 4. Wound-Healing Evaluation Parameters

### 4.1. Measurement of Wound Area

The wound areas were traced on transparency paper each two days. The wound surface areas were then measured using a software application (AutoCAD 2015) for design and drafting.

Wound contraction, expressed as a reduction of the original wound size, was calculated using the following equation:

(1) $$\begin{array}{c} \text{Wound closure}\;\left(\;\%\;\right)=\left[\;\frac{\left(A_{0}-A_{d}\right)}{A_{0}}\;\right]\times 100, \end{array}$$

where *A*_0_ and *A*_*d*_ are the initial wound area (day 0: wounding day) and wound area on day (d), respectively.

### 4.2. Chromatic Study

A chromatic code was attributed to each wound as follows: bright red = blood covering the wound; dark red = coagulation of blood in the epidermis; red = granulation tissue; and pink = epithelialization step [24].

### 4.3. Wound Contraction and Epithelialization Time

This was estimated by observing the number of days necessary for the scab to drop off from the wound surface [25].

### 4.4. Hydroxyproline Estimation

The estimation of hydroxyproline content was achieved according to Bergman and Loxley [26] and results were reported as mg/g dry weight of tissue.

### 4.5. Histological Examination

Tissue specimen samples from wound site of all studied groups were tacked and fixed in 10% neutral buffered formalin solution, embedded in paraffin wax, and cut and stained with hematoxylin and eosin. The sections were observed under a light microscope regarding fibroblast proliferation, collagen formation, angiogenesis, and epithelialization.

### 4.6. Statistical Analysis

Data were expressed as mean values ± standard deviation (SD). A statistical significance comparison between groups was accomplished using the SPSS version 20.

The mean differences between the different groups were assessed by Duncan test and compared by one-way analysis of variance (ANOVA). Differences were considered significant at *P* < 0.05.

## 5. Results

### 5.1. Phytochemical Screening and Antioxidant Activity of* U. dioica*

Total phenolic and flavonoid contents and the antioxidant activity of the different* U. dioica* organic extracts are shown in Table 1. Phytochemical screening of EtOH-H_2_OE of* U. dioica* leaves and their fractions showed that alkaloids, tannins, polyphenols, and flavonoids are more abundant in the EtOH-H_2_OE than the leave fractions. Indeed, the EtOH-H_2_OE had the highest amount of phenolic compounds (116.9 ± 5.416 mg GAE/g) and flavonoids (43 ± 0.019 mg EQ/g) followed by the EtOAc-F (23.15 mg GAE/g and 33.2 mg EQ/g, resp.). The increased amount of polyphenolic compounds in the EtOH-H_2_OE was coupled with the best antioxidant activity in the DPPH and *β*-carotene bleaching test assays. EtOH-H_2_OE of* U. dioica* showed DPPH scavenging activity with a prominent IC_50_ value of 10.4 *μ*g/mL when compared to ascorbic acid (3.5 *μ*g/mL) a well-known antioxidant. The oxidation was effectively inhibited, in the *β*-carotene linoleic acid assay, by the EtOH-H_2_O plant extract. The IC_50_ value was 15 *μ*g/mL compared to BHT (5 *μ*g/mL) taken as reference.

### 5.2. *U. dioica* EtOH-H_2_OE GC-MS Analysis

The fatty acids (FAs) results and sterol composition were depicted in Table 2. Numerous fatty acids were detected in EtOH-H_2_OE of* U. dioica*. The main identified polyunsaturated fatty acids were palmitoleic (C_16:1_), oleic (C_18:1_), linoleic (C_18:2_), and linolenic (C_18:3_).

The EtOH-H_2_OE of* U. dioica* leaves analysis revealed the presence of a high amount of polyunsaturated fatty acids known as essential fatty acids (37.85% of the total FAs). Palmitic acid, the most common saturated fatty acid and the precursor of unsaturated fatty acids, was present at a considerable rate (13.53%). In Table 2, lupeol, a triterpene, accounted for about 86% of the total sterols.

### 5.3. Antibacterial Activity

The* U. dioica organic *extracts antibacterial activity was qualitatively assessed against tested microorganisms (two Gram-negative and two Gram-positive bacteria species) according to inhibition zones and the determination of the Minimum Inhibitory Concentrations (MIC). The results are represented in Table 3.

Among the tested extracts, only EtOH-H_2_OE and EtOAc-F exhibited an antibacterial activity. The best activities were recorded against* Pseudomonas aeruginosa* (20 ± 0.2 mm) and* Enterococcus faecalis* (18 ± 0.6 mm) in the EtOH-H_2_OE with the lowest MIC values of 0.312 and 1.25 mg/mL, respectively.* n*-Hexane and water fractions remained inactive in the range of the studied concentrations (2.5 mg/wells).

### 5.4. Bleeding Time in Rats

The bleeding time in rats' data revealed that the best reduction was observed with EtOH-H_2_OE of* U. dioica*. According to this test, the bleeding time significantly decreased (*P* < 0.001) in the group treated with EtOH-H_2_OE (4.83 ± 0.44 sec) compared to the untreated group (112.66 ± 2.51 sec) and positive control group (36 ± 2.2 sec).

### 5.5. Wound-Healing Evaluation Parameters

#### 5.5.1. Body Weight

Body weight changes are listed in Table 4. Our findings revealed an insignificant increase of rats' weight after treatment in all the studied groups. However, these disparities did not reveal a difference in the mean body weight between all groups of rats at the end of the experimental period.

#### 5.5.2. Chromatic Study

The wound-healing activity was assessed, each two days, by a chromatic study based on the progressive changes in wound color during the different wound-healing phases. The photos of the wound appearance, of the four tested groups, are illustrated in Table 7.

The chromatic study showed similar coloration of the wound in the first 2 days for the four studied groups: a bright red color (corresponding to the blood covering the wound) during the first day and a dark red color in the second day. By the third day, the untreated and glycerol treated groups showed a large inflammatory rim around the injuries with increased amounts of exudates. From the third day, a brown color scab was detected in the* U. dioica* treated group and towards day 7 for “CICAFLORA” treated group. This scab persisted in all the treated groups from day 7 to day 9 of the experiment. By day 9, an erythema around the damaged skin of the untreated wounds was shown.

After 11 days, scabs had exfoliated in the* U. dioica* treated group showing pink blade coloration but the reference drug group wounds persisted to be open and showed just residual scab. However, these scabs still persisted in the untreated and glycerol treated groups and showed open wounds.

#### 5.5.3. Assessment of Wound Closure

The healing process was examined during the 11-day experimental period to appraise the wound-healing ability of the tested extract by following the wound closure rate. The results were compared to “CICAFLORA cream” a standard reference drug. This repairing drug contains 10% of* Mimosa tenuiflora* extract as the main active component. This rate was evaluated for each group by determining the closed injuries as a function of time. The rate measure of the wound closure of all groups is presented in Table 5.

As expected, significant wound-healing activity was showed in CICAFLORA and* U. dioica* treated groups compared with glycerol and control groups (Table 5). The untreated group showed the lower wound closure than the treated ones (*P* < 0.001). The rate of wound closure in the rats treated with EtOH-H_2_OE of* U. dioica* was 74.45% on 9th day and 92.39% on 11th day, respectively. As shown in Table 5, the untreated wounds healed much more slowly.

At the end of experiment, by day 11 of the treatment, almost all the wounds treated with* U. dioica* showed complete wound closure (92.39%) against only 85.36% of wound healing recorded in CICAFLORA. The untreated rats still exhibited unhealed open injuries at the end of the experiment (60.91%). Thus, the mean time taken for reepithelization of the excision wound in EtOH-H_2_OE of* U. dioica *treated group was less compared to the animals treated with the reference drug.

#### 5.5.4. Estimation of Hydroxyproline Content

Hydroxyproline is an amino acid specific of collagen. As reported in Table 6, the hydroxyproline level increased in the group treated with* U. dioica* (25.9 mg/100 mg of tissue) when compared to the untreated group (18.97 mg/100 mg of tissue) and CICAFLORA (22.68 mg/100 mg of tissue) treated groups (*P* < 0.05).

#### 5.5.5. Histopathological Study

**Figure 1:**
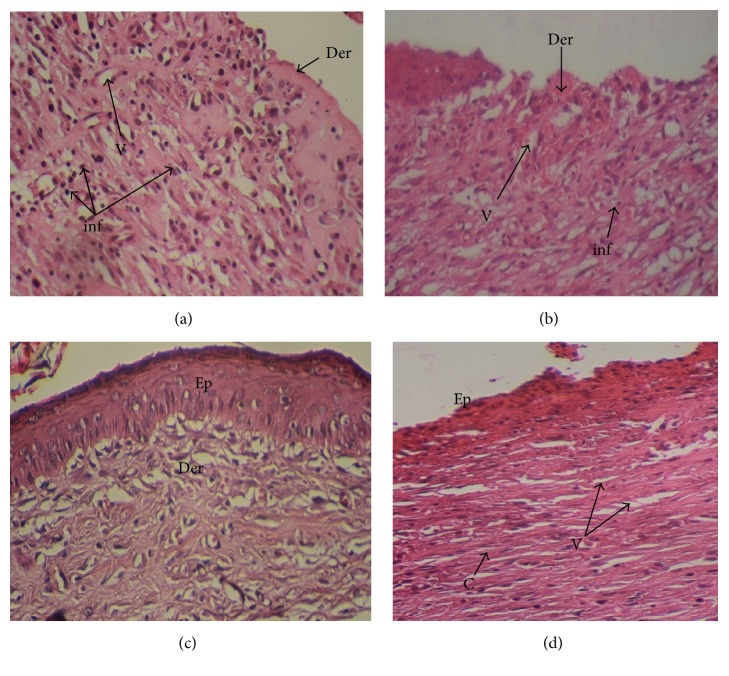
The representative photomicrographs of the effect of the physiological serum (a); glycerol (b);* U. dioica* (c); and “CICAFLORA” (d) treatment on rat, showing epidermal and dermal architecture of wounds on the 11th day. HE-stained histological sections are 5 mm thick and photomicrographs are taken at 20x magnifications. Ep: epidermis; Der: dermis; V: blood vessels; inf: inflammatory cells; C: collagen.

On the last day of treatment (day 11) histological observations were performed on samples. They are displayed in Figure 1.

The biopsies from tissues were evaluated for reepithelization, revascularizations, fibroblasts, and inflammatory cells. The histological study of the granulation tissue of the untreated and glycerol treated groups showed incomplete epithelial regeneration (Figures 1(a) and 1(b)), respectively. Further, numerous inflammatory cells and many immature fibroblasts were shown in the untreated groups. However, biopsies from the* U. dioica* treated rats (Figure 1(c)) showed significant neovascularization, a clearly identified thick epidermis, and fibroblasts. The boundary layer between the epidermis and the dermis was clear. Biopsies from the reference group “CICAFLORA” showed no epidermal layer or an incomplete one and considerable inflammatory cells (Figure 1(d)). Also, the presence of fibroblasts in the dermis indicated completion of healing. The reference drug and the control groups exhibited delayed wound-healing process compared to that of* U. dioica* treated group.

## 6. Discussion

Plants have rich medicinal value and potential sources of new wound-healing compounds. Nowadays, plant extracts are viewed as an efficient alternative for wound healing thanks to their widespread availability. The wound healing is a highly complex mechanism of repair following injury. During the healing process, an inflammatory response occurs leading to the generation of ROS well known for their noxious effect. Furthermore, an infection by* S. aureus* and* P. aeruginosa* can delay the inflammatory phase and disrupt the normal clotting system hence delaying the angiogenesis. The improved wound-healing effects of various plant extracts may be attributed to the antioxidant and antimicrobial properties of their phytoconstituents. A positive role has been shown between free radical-scavenging action and wound-healing process [27]. In the current study, the EtOH-H2OE of* U. dioica* was found to have remarkable antioxidant and antibacterial activities* in vitro*. The phytochemical analysis of EtOH-H_2_OE of* U. dioica *showed the richness of this extract in phenolic and flavonoid compounds. The positive correlation between total phenolics content and antioxidant activity (*R*^2^ = 0.96) of* U. dioica *extract explains the ROS scavenging capacity [28]. Furthermore, the* U. dioica *extract exerts antibacterial effects, against Gram-positive and negative bacteria such as* S. aureus *and* P. aeruginosa *known as the commonest strains associated with open wound infections [29]. The richness of* U. dioica* EtOH-H2OE in phenolics, known for their powerful antioxidant and antibacterial properties, also involved in modulation of hemostasis and reduction of healing time [30–32], prompted us to assess the homeostatic and wound-healing effects of* U. dioica *on rat skin wound-healing model. According to the primary homeostasis test, the extract has significantly (*P* < 0.001) shortened the bleeding time by boosting blood clotting. This result suggests that this extract contains some phytochemical compounds with antiplatelet and fibrinolytic activities which could contribute to the healing process [33].

The investigation of the rats' body weights of the studied groups during the experimental period showed their normal growth suggesting that the used extract had no side effects on rats. The morphometric assessment showed increased contraction rates of the wounds from the treated groups than in the untreated ones. Additionally, wound area decreased significantly in* U. dioica* treated group on day 11 (*P* < 0.01). Previous research using the same wound model and ethanolic extract of* Sida cordifolia* has reported that complete wound closure was observed within 14 days [34]. However, it is clearly observed that it was shortened to 11 days in the current study.

Visual observations revealed that granulation tissue was observed earlier in the treated injuries compared to the control ones. This proves that topical use of* U. dioica* extract was found to be equivalent to the reference cream in the advancement of granulation tissue formation. The histological study of granulation tissue of untreated animals demonstrated an invasive macrophage migration with limited collagen fibers compared to the treated ones. However, healed biopsies of* U. dioica *treated rats exhibited a granulation tissue with a significant increase in collagen content with more fibroblasts, small new blood vessel, and fewer inflammatory cells. A significant enhancement in collagen deposition was observed during the wound-healing process in* U. dioica* treated group due to the increased migration of fibroblasts and epithelial cells to the wound area. The reduced collagen density in the untreated group might be due to a persistent inflammatory stage where the degradation of collagen will exceed its synthesis [35]. Moreover, a higher level of hydroxyproline, a molecule involved in collagen formation and wound healing, was revealed in* U. dioica* treated group compared to that in the untreated group. This implies an increased collagen turnover.

Moreover, the tested extract showed the presence of alkaloids which are involved in reepithelialization process, a crucial step towards wound healing [36]. The richness of* U. dioica* extract in alkaloids, tannins, polyphenols, and flavonoids may also synergistically promote angiogenesis. Hoeben et al. [37] have previously reported a significant relationship between phytochemical composition of plant extract and the neoformation of microvessels in the wound-healing process. It was also reported that polyphenols can regulate angiogenesis* in vivo* and proliferation of epithelial cells* in vitro *[38]. All these findings are in agreement with the histological observations after topical application of the tested extract with thin epithelium, organized collagen fibers, and omitted inflammatory cells. Interestingly, the morphological evaluation on day 11 revealed that the wounds treated with* U. dioica* were relatively clean and free from any inflammatory reaction compared to the negative control groups with damaged skin and pus accumulation. Several studies have previously demonstrated the wound-healing activity of medicinal plants. The extract of* Ocimum sanctum* significantly increased the wound breaking strength by faster epithelialization and increased wound contraction rate [39]. Ammar et al. [40] showed that wounds treated with methanolic extract of* Opuntia ficus-indica* contain more collagen deposition and fewer inflammatory cells and angiogenesis which were in accordance with our data. Our results revealed that the* U. dioica *treated group healed faster and showed less scarring, more blood capillaries, less inflammatory cells, and more fibroblasts and collagen. Thus, the* U. dioica *enhances the wound-healing process by increasing the rate of various phases, such as cell proliferation, angiogenesis, and collagen formation.

The GC-MS analysis of EtOH-H_2_OE of* U. dioica *showed the highest level of lupeol in this extract. Previous reports have evaluated the efficiency of lupeol in the wound-healing process and reported that lupeol isolated from the* Celastrus paniculatus* extract improved reepithelization, with an enhancement of granulation tissue and collagen [41]. Moreover, recent observations highlighted the anti-inflammatory effects of phytosterols which can stimulate new skin growth by attracting macrophages and enhancing fibroblast and collagen deposition [42]. On the other hand, the tested extract contained a significant amount of unsaturated fatty acids (37.85%) such as the palmitoleic, oleic, linoleic, and linolenic acids. It was stated that fatty acids agents accelerate the healing of wounds thanks to their proinflammatory effect [43]. Thus, they increase angiogenesis, cell proliferation, and collagen formation and contribute to the antibacterial activity [44].

Wound-healing efficacy of* U. dioica *EtOH-H_2_OE observed in this work originates most probably from a synergistic interaction between its antioxidant capacity and antibacterial property owing to its richness in various effective chemical compounds present in the extract such as phenolics, fatty acids, and lupeol.

## 7. Conclusion

The present study revealed that the hydroalcoholic extract obtained from* U. dioica* has a significant wound-healing activity which was evident by the increased rate of wound contraction and reduction in the period of epithelialization and angiogenesis. The total extract richness in various chemical compounds and the antioxidant and antibacterial capacities clearly contributed to its medicinal properties. However, the identification and elucidation of active components in this plant may provide useful knowledge and lead to the development of new and effective drugs against wounds. The combination of traditional and modern knowledge can produce better drugs for wound healing with fewer side effects. The results obviously highlight the health uses of this traditional plant for future clinical applications.

## Figures and Tables

**Table 1:** Amounts of total phenolic compounds, total flavonoids, and determined IC_50_ values of the DPPH free radical-scavenging assay and *β*-carotene bleaching test of *U. dioica*. Ascorbic acid and BHT were used as standards.

Extract	Phenolic content^a^ (mg GAE/g)^b^	Flavonoids content^a^ (mg EQ/g)^c^	DPPH^a^ (IC_50_ *µ*g/ml)	*β*-Carotene^a^ (IC_50_ *µ*g/ml)
EtOH-H_2_OE	116.9 ± 5.416	43 ± 0.019	10.4 ± 0.001	15 ± 0.001
*n*. Hex-F	ND	ND	206 ± 0.106	210 ± 0,001
EtOAc-F	23.15 ± 2,674	33.2 ± 0,033	198 ± 0.054	175 ± 0.001
Water-F	8.8 ± 1.442	9.96 ± 0,018	286 ± 0.002	243 ± 0.001
Ascorbic acid	—	—	3.5 ± 0.2	—
BHT	—	—	—	5.1 ± 0.1

^a^Each value represents the mean ± SD of three experiments; ^b^: mg of gallic acid equivalent per g of dry plant extract; ^c^: mg of Quercetin equivalent per g of dry plant extract; ND: not detected; —: not tested.

**Table 2:** GC/MS analysis of fatty acids, terpenes, and sterols of the *U. dioica *EtOH-H_2_OE.

Peak	Components	Fatty acids (FAs)	Retention time (min)	Relative composition (%)
			Saturated fatty acids (SFAs)	
1	Myristic acid	C14:0	26.067	0.39
2	Pentadecanoic acid	C15:0	28.946	1.37
3	Palmitic acid	C16:0	29.707	13.53
5	Stearic acid	C18:0	32.337	13.79
9	Arachidic acid	C20:0	36.247	0.64
10	Behenic acid	C22:0	39.209	0.30
	∑ saturated fatty acids			30.02

			Unsaturated fatty acids (SFAs)	
4	Palmitoleic acid	C16:1 n-7	30.182	1.29
6	Oleic acid	C18:1 n-9	32.676	24.33
7	linoleic acid	C18:2 n-6	33.301	10.4
8	Linolenic acid	C18:3 n-3	34.154	1.83
	∑ unsaturated Fatty acids			37.85
	Total fatty acids			67.87
		Esters of FAs		
11	*β*-Glyceryl oleate	C18:1 n-9	37.016	0.44
12	*α*-Glyceryl oleate	C18:1 n-9	37.122	0.14
	∑ esters FAs			0.58
	Ni-*compounds*^a^			31.55

		Terpenes and sterols		
1	Lupeol	—	25.72	85.96
2	Neophytadiene	—	26.361	0.14
3	Phytol	—	31.809	0.95
4	Stigma-4-en-3-one	—	46. 631	0.09
5	*β*-Sitosterol	—	47.000	0.24
	Total compounds			87.38
	Ni-*compounds*^a^			12.62

^a^Total of nonidentified compounds.

**Table 3:** Antibacterial activity of organic extracts of *U. dioica *(inhibition diameter ZI in mm and MIC in mg/mL).

	EtOH-H_2_OE		EtOAc-F		Gentamicin
	ZI	MIC	ZI	MIC	ZI
*S. aureus ATCC 25923*	12 ± 0.1	5	10 ± 0.4	5	25 ± 0.8
*E. faecalis* ATCC29212	18 ± 0.6	1.25	12 ± 0.8	2.5	20 ± 0.2
*P. aeruginosa ATCC 27853*	20 ± 0.2	0.312	0	ND	18 ± 0.5
*E. coli ATCC 25922*	0	ND	0	ND	21 ± 0.9

Values are given as mean ± SD of triplicate experiment. Gentamicin was used as a standard antibiotic at a concentration of 15 *µ*g/well; ND: not determined.

**Table 4:** Variation of the body weights of rats among the experimental period.

Day	Group I	Group II	Group III	Group IV
Before treatment	162 ± 1.58	163 ± 4.39	169 ± 6.16	161 ± 4.51
After treatment	173 ± 3.66	173.8 ± 4.02	178 ± 5.89	174 ± 6.21

Data are expressed as mean ± SD for weight of six rats in each group. Means followed by the same column are not significantly different at *P* > 0.05. Group I was treated with physiologic serum (negative control); Group II was treated with glycerol; Group III was treated with “CICAFLORA cream” (positive control); and Group IV was treated with the hydroalcoholic extract of *U. dioica.*

**Table 5:** Effects of *Urtica dioica* and CICAFLORA on wound contraction (%).

Days	0	3	5	7	9	11
Group I	0.00	3.50 ± 0.14^c^	7.19 ± 1.13^d^	19.63 ± 0.34^d^	30.63 ± 1.49^d^	60.91 ± 1.16^d^
Group II	0.00	9.22 ± 0.68^b^	16.62 ± 1.13^c^	32.15 ± 0.84^c^	49.73 ± 1.17^c^	70.30 ± 0.31^c^
Group III	0.00	8.33 ± 1.13^b^	31.56 ± 1.17^b^	42.36 ± 0.35^b^	62.18 ± 0.44^b^	85.36 ± 1.96^b^
Group IV	0.00	12.36 ± 0.21^a^	34.01 ± 1.14^a^	48.97 ± 1.14^a^	74.45 ± 0.94^a^	92.39 ± 1.13^a^

Values are given as mean standard deviation for groups of six rats each. Data with different letters for each column represent significant difference at *P* < 0.05. Group I was treated with the physiologic serum (negative control); Group II was treated with glycerol; Group III was treated with “CICAFLORA cream” (positive control); and Group IV was treated with the hydroalcoholic extract of *Urtica dioica*.

**Table 6:** Hydroxyproline content in different experimental animal groups. All values are mean ± SD (*n* = 6/group).

Groups	Hydroxyproline mg/100 mg of tissue
Group I	18.97 ± 0.1^c^
Group II	18.2 ± 0.3
Group III	22.68 ± 0.21^b^
Group VI	25.90 ± 0.45^a^

Group I was treated with physiologic serum (negative control); Group II was treated with glycerol; Group III was treated with “CICAFLORA cream” (positive control); and Group IV was treated with the hydroalcoholic extract of *U. dioica.*^a, b, c^Different letters in the same column indicate significant differences (a > b > c; *P* < 0.05).

**Table 7:** Representative photographs of macroscopic assessment of wounds for the four studied groups on day 1, day 3, day 7, and day 11.

Days	Group I: untreated group	Group II: glycerol treated group	Group III CICAFLORA treated group	Group IV: *U. dioica* treated group
Day 1	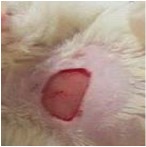	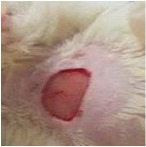	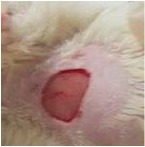	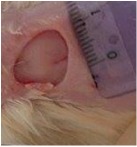
Day 3	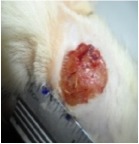	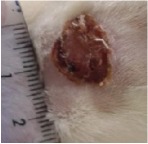	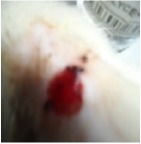	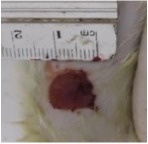
Day 7	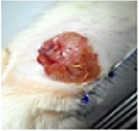	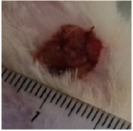	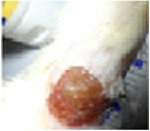	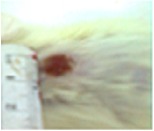
Day 9	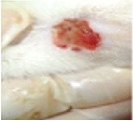	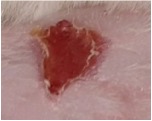	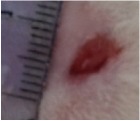	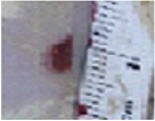
Day 11	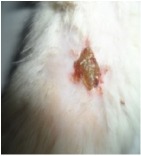	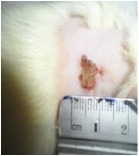	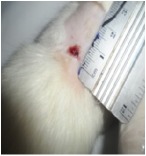	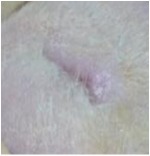

Group I was treated with physiologic serum (negative control); Group II was treated with glycerol; Group III was treated with “CICAFLORA cream” (positive control); and Group IV was treated with the hydroalcoholic extract of *U. dioica*.
